# Honey Bee Survival and Pathogen Prevalence: From the Perspective of Landscape and Exposure to Pesticides

**DOI:** 10.3390/insects9020065

**Published:** 2018-06-13

**Authors:** Mohamed Alburaki, Deniz Chen, John A. Skinner, William G. Meikle, David R. Tarpy, John Adamczyk, Scott D. Stewart

**Affiliations:** 1 West Tennessee Research and Education Center, Department of Entomology and Plant Pathology, The University of Tennessee, Jackson, TN 38301, USA; sdstewart@utk.edu; 2 Department of Biological Sciences, the University of Southern Mississippi, Hattiesburg, MS 39406, USA; 3 Department of Entomology and Plant Pathology, North Carolina State University, Raleigh, NC 27695-7613, USA; deniz.m.chen@gmail.com (D.C.); drtarpy@ncsu.edu (D.R.T.); 4 Department of Entomology and Plant Pathology, The University of Tennessee, Knoxville, TN 37996, USA; jskinner@utk.edu; 5 USDA-ARS Carl Hayden Bee Research Center, Tucson, AZ 85719, USA; william.meikle@ars.usda.gov; 6 USDA-ARS—Thad Cochran Southern Horticultural Laboratory, Poplarville, MS 39470, USA; john.adamczyk@ars.usda.gov

**Keywords:** agricultural landscape, crops, honey bees, pesticides, pollinator, varroa, virus

## Abstract

In order to study the in situ effects of the agricultural landscape and exposure to pesticides on honey bee health, sixteen honey bee colonies were placed in four different agricultural landscapes. Those landscapes were three agricultural areas with varying levels of agricultural intensity (AG areas) and one non-agricultural area (NAG area). Colonies were monitored for different pathogen prevalence and pesticide residues over a period of one year. RT-qPCR was used to study the prevalence of seven different honey bee viruses as well as *Nosema* sp. in colonies located in different agricultural systems with various intensities of soybean, corn, sorghum, and cotton production. Populations of the parasitic mite *Varroa destructor* were also extensively monitored. Comprehensive MS-LC pesticide residue analyses were performed on samples of wax, honey, foragers, winter bees, dead bees, and crop flowers for each apiary and location. A significantly higher level of varroa loads were recorded in colonies of the AG areas, but this at least partly correlated with increased colony size and did not necessarily result from exposure to pesticides. Infections of two viruses (deformed wing virus genotype a (DWVa) and acute bee paralysis virus (ABPV)) and *Nosema* sp. varied among the four studied locations. The urban location significantly elevated colony pathogen loads, while AG locations significantly benefited and increased the colony weight gain. Cotton and sorghum flowers contained high concentrations of insecticide including neonicotinoids, while soybean and corn had less pesticide residues. Several events of pesticide toxicity were recorded in the AG areas, and high concentrations of neonicotinoid insecticides were detected in dead bees.

## 1. Introduction

Recent increases in honey bee (*Apis mellifera*) mortality and other pollinating insects have been described as multifactorial [1,2,3]. However, some factors seem to be more directly linked to honey bee loss than others. Parasitism by varroa mite *Varroa destructor* is considered a major contributor to the collapse of honey bee colonies [4,5,6]. Other contributing factors include viral and bacterial infections, poor nutrition, exposure to chemicals used for in-hive pest control, and other agricultural pesticides that bees encountered while foraging [7,8,9]. Varroa is an external parasite that affects both adult bees and brood by sucking their hemolymph, which weakens and shortens the honey bee’s life and creates various type of deformation in emerging brood [6,10,11]. Furthermore, varroa mite is known to be a very efficient disease vector, particularly of viruses, and its control generally requires chemical treatment [2,12]. Besides the large spectrum of agricultural pesticides used for pest crop control, it should be considered that miticides applied in-hive to treat varroa mite can threaten colony health [13,14]. Multiple chemical residues, such as coumaphos and fluvalinate, resulting from varroa treatment are often detected inside bee hives, especially in the beeswax [15,16]. A recent study showed that continuous accumulation of pesticide residues in beeswax, more particularly fungicides, appear linked to colony mortality [17]. The accumulation through the time of sublethal pesticide concentrations in the hive may contribute to progressive decline in the bee population of a colony. It has recently been demonstrated that sublethal doses of neonicotinoids reduce male production [18], impair the olfactory memory and learning capacity of worker bees [19,20] and alter the homing behavior of forager bees [21].

In addition to varroa, viruses and nosema, honey bee colonies are also exposed to other stressors such as pesticides and variable forage [22]. The quality and quantity of the accessible forage surrounding bees, as well as the type of the landscape, have a major importance on their survival and performance [23,24,25,26]. In this study, honey bee colonies were studied for pathogen prevalence in three different landscapes with varying intensities of agriculture (AG areas) and one non-agricultural area (NAG area). It however remains challenging while conducted honey bee in situ studies to judge or evaluate each factor individually, as most factors affecting colony health act in complex synergistic manners with variable level implications [2,27,28,29,30,31]. Many studies have shown synergetic effects of exposure to pesticides and honey bee pathogens [28,32], in particular varroa mite infestation levels [32]. Others described positive correlations among pests and pathogens such as varroa, viruses and nosema [2,33,34], which clearly reduces the probability of identifying the first factor that triggered the colony decline. Therefore, a continuous monitoring of the colony health and performance throughout the experiment and thorough screening of various pathogens at different time points might provide more precision on the main factor or factors that initiate the decline of a colony.

This study is a continuation of a previous work [26] and was conducted on the same honey bee hives and locations. The current work was intended to test how differences in exposure to pesticides and landscape may influence the levels of various pathogens in honey bee colonies, as well as the residual levels of pesticides in hive products, dead bees, and foragers. Possible links and interactions between exposure to pesticides, landscapes, and pathogens were evaluated in order to explain the differences obtained in colony health and survival.

## 2. Materials and Methods

### 2.1. Location and Landscape

The experimental locations (Jackson, Milan, Yum-Yum, and Chickasaw) were chosen based on a typical 2.5 km-radius foraging distance for honey bees [35] and potential exposure to pesticides and crop intensity. Geographical information system studies GIS was conducted using Esri ArcGIS^®^ software [36] as described in [26].

### 2.2. Honey Bee Colonies

This experiment was conducted on sixteen honey bee colonies headed by Carniolan (*Apis mellifera carnica*) queens as described in [26]. A board (1.5 × 1.5 m) was placed in front of each hive to help observe and collect any bee mortality at the front entrance. When an unusual number of dead bees (~50 or more) were observed on those boards, they were collected and sent for pesticide residue analysis.

### 2.3. Varroa Mite Infestation

All experimental honey bee hives were equipped with varroa mite screen boards in order to perform varroa mite counts. Passive varroa mite counts were made biweekly from those boards for 72 h from May 2015 to April 2016 (Table 1). In total, twenty reads were recorded for each colony including the winter time period. Hives were treated one time for varroa mite with amitraz (October 2015) using two strips of Apivar^®^ per hive (Table 1).

### 2.4. Viral and Nosema Infections

#### 2.4.1. Sampling

A hundred worker bees per colony were sampled monthly during the period of bee activity (May–October) to study the levels of seven viral infections in our experimental colonies. The studied viruses were: deformed wing virus genotype a (DWVa), deformed wing virus genotype b (DWVb), black queen cell virus (BQCV), acute bee paralysis virus (ABPV), sac brood virus (SBV), Lake Sinai virus (LSV), and chronic bee paralysis virus (CBPV). Level of *Nosema* sp. infestation was also quantified in all worker samples by quantitative PCR using universal primers designed to detect both nosema species (*N. ceranae* and *N. apis*) [37,38]. Worker bees were sampled from the brood nests and killed on dry ice until arriving to the lab and stored at −80 °C. In total, 96 worker bee samples were analyzed for viral and nosema infections.

#### 2.4.2. RNA Extraction

Total RNA was extracted from the whole worker’s body using TRIzol^®^ Reagent protocol from Invitrogen [38] with some modifications. Fifty workers were randomly selected from each sample and put in a sterile plastic bag with 5 mL Trizol and gently crushed and mixed for 2 min. One mL of that mix was transferred to a 2 mL fresh tube and 200 μL of chloroform was added. The total mixture was incubated at room temperature for 15 min followed by a centrifugation at 10,200 rpm for 15 min at 4 °C.

Three hundred μL of the supernatant was transferred to a fresh tube, and the tube was washed with 500 μL each of isopropanol and incubated for 15 min at room temperature, followed by centrifugation at 10,200 rpm for 15 min at 4 °C. The pellet was subsequently washed with 1 mL 75% ethanol and centrifuged at 10,200 rpm for 15 min at 4 °C. Finally, the RNA pellet was well dried and 60 μL of nuclease-free water was added. RNA extractions were nanodropped (Thermo Scientific NanoDrop 2000/2000c Spectrophotometers, Waltham, MA, USA) for RNA quantity and quality and were diluted to 200 ng/μL and stored at −80 °C.

#### 2.4.3. RT-qPCR Steps

Two-step reverse transcription quantitative PCR (RT-qPCR) was used to quantify the viral loads of each studied virus as well as *Nosema* sp. in our bee samples. One μg of RNA was used as a template for cDNA synthesis using BioBasic High Reverse Transcriptase kits and random hexamer primers. RT-qPCR was performed, in triplicate, on a BioRad CFX384 using LifeTechnologies PowerUP SYBR Green master mix. The viral genes were normalized using GeNorm [39] in all the RT-qPCR runs using a set of four reference genes (Actin, CamIIk, Apo28S, and Ancr1). All primers’ sequences used in this study are available in the DOI in the supplementary information.

### 2.5. Detection of Pesticide Residues

Pesticide residues were quantified in forager bees, honey, wax, dead bees collected from hives’ entrances, winter bees, and flowers haphazardly collected from corn, cotton, soybean, and sorghum in surrounding agricultural fields using liquid chromatography-mass spectrometry (LC-MS) [38]. All the chemical analyses for pesticide residue detection were processed at the USDA National Scientific Laboratories in Gastonia, North Carolina. A comprehensive chemical analysis that included 174 chemical substances was run for each sample, and positive results were reported in the text. Complete analytical reports can be found in the DOI in the supplementary information.

#### 2.5.1. Forager Bees

Traps were designed to sample foragers at the hive entrance during their return flight home (see DOI/Supplementary Information). A metallic screen box (15 × 2 × 40) cm opened from one side (40 cm) was built for this purpose. The screen box was slipped into the hive entrance for 15 cm and completely blocked the entrance allowing only returning-foragers to access the box cavity from the hive entrance. Once a significant number of foragers were trapped, the screen box was gently removed from the entrance and foragers killed on dry ice and later on stored at −80 °C. Foragers were sampled monthly from each hive from May to September 2015 (Table 1). Foragers of each apiary (location) were pooled and 20 samples in total were sent for chemical analysis. The number of bees in each analyzed sample was ~1000 foragers.

#### 2.5.2. Honey and Wax

Approximately 10–15 mL of freshly collected nectar/honey were sampled from 2–3 frames per hive from June to September 2015 (Table 1). Honey collected from each apiary at each date was pooled and stored at −80 °C. In total, 16 honey samples were sent for chemical analysis. Wax was only sampled once from freshly built combs at the beginning of the study (May 2015). Samples of each apiary were pooled and also stored at −80 °C. In total, four wax samples (one from each apiary) were sent for pesticide chemical residue analysis.

#### 2.5.3. Winter and Dead Bees

In order to assess prolonged pesticide persistence in bees, a set of winter bees were sampled at the end of the wintering (March 2016) and sent for chemical analysis. Hundreds of winter bees were sampled from the bee nest cluster of each colony, samples of each apiary were pooled, and four samples were sent for chemical analysis (Table 1). During the summer months, considerable numbers of dead bees (>50) were observed five times on the boards fixed in front of each hive. Dead bees were collected from those boards and sent for chemical analysis.

#### 2.5.4. Crop Flowers

Flowers of the main four crops (cotton, soybean, corn, sorghum) growing around the experimental apiaries were sampled and analyzed for pesticide residues. During the blooming period of each crop, flowers were sampled from the closest and biggest fields (3–5 fields) around the hives, with an average of 10–20 sample points per field. Sampled flowers of each location and each crop type were pooled. In total, 12 samples of crop flowers were analyzed from the four crop flowers around the three apiaries located in the AG areas.

### 2.6. Statistical Analysis

Statistical analyses and figure generation were carried out and generated in the R environment, version 1.1.419—© 2008—2018 RStudio software, (Boston, MA, USA). [40]. Data were mainly treated per location (four groups) to study the landscape effects and potential exposure to pesticides on honey bee colony health. In some cases, data were analyzed by two groups (AG and NAG) areas to explore the putative impact of the cropping intensity on the honey bee colonies.

Variables of this study included: (1) percentage of the agricultural area in each location; (2) number of counted varroa per colony and date (varroa infestation); (3) prevalence of seven viruses (viral infection); and (4) level of nosema infection. Viral and nosema results are obtained in relative quantification (RQ). Each variable was tested for normality by Shapiro-Wilk test and log-transformation failed to normalise the distribution of the data as shown in the Q-Q plots (Figure S4). Since the analysis of variance (ANOVA) is not very sensitive to moderate deviations from normality [41,42,43], it was carried out at a 95% confidence level. Correlations between variables (viral load vs. varroa infestation and colony weight vs. varroa infestation) were performed using the R libraries “PerformanceAnalytics” and “corrplot”, respectively. Colony weight data used in this study in the correlations analyses is part of the same project and was previously published [26]. Generalized linear mixed models GLM were used to assess the accumulative effect of varroa load, viral and nosema infections and were carried out as described in our previous study [26].

## 3. Results

### 3.1. Landscape Study

**Figure 1:**
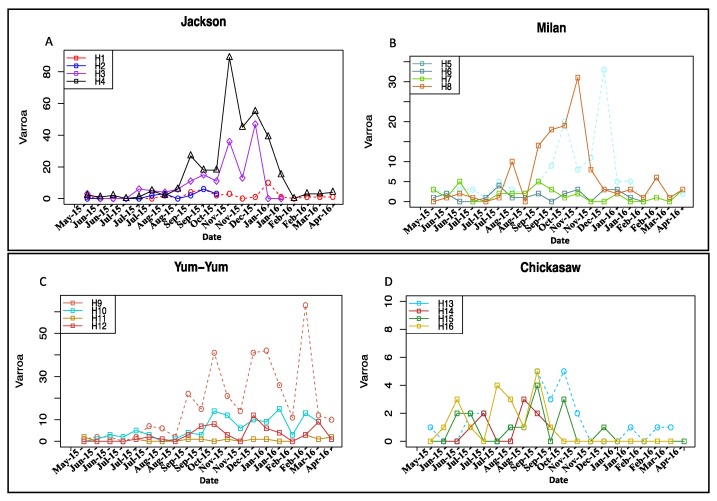
Number of varroa mite exposed per colony and date for each of the sixteen studied colonies. Varroa loads were biweekly counted using sticky mite board fixed at the bottom hive. (**A**) Group colonies of Jackson; (**B**) for those of Milan; (**C**) Yum-Yum; and (**D**) for colonies of Chickasaw.

The locations and their agricultural classification based on the GIS were as described in [26]. Briefly, Jackson: a low AG area with urban activity, Milan: moderate AG area, Yum-Yum: high AG area and Chickasaw: a natural park that contains no agricultural activity; NAG area. For more details on the location sites and landscape composition refer to Figure 1 and Table 1 of our previously published study [26].

### 3.2. Varroa Infestation

**Figure 2:**
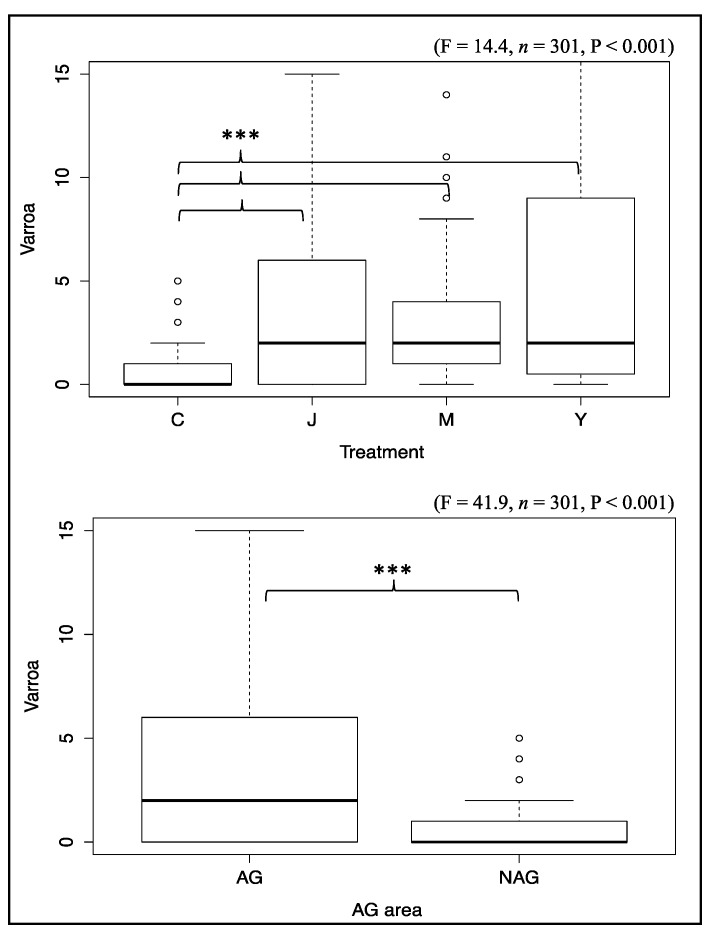
Overall level of varroa infestation exposed by treatment and AG area. Abbreviation codes are (C) Chickasaw, (J) Jackson, (M) Milan, and (Y) Yum-Yum. The Boxplots (aka, Box and whisker Plots) summarize the data distribution based on minimum, first quartile, median, third quartile, and maximum values. Highly significant differences (*p* < 0.001) were found in varroa load between groups vis-à-vis the treatment and AG area factors. ANOVA signification levels are *** *p* < 0.001.

Levels of varroa loads varied significantly among locations and hives (Figure 1). The highest varroa counts were mainly observed in the fall and winter seasons (September–March) (Figure 1) except for colonies of Chickasaw (Figure 1D). The highest number of varroa observed in this study was located in Jackson where 89 varroa were found on the sample board counted on November 9 (Hive 4) (Figure 1A). Overall varroa load was significantly lower in colonies of Chickasaw location (Figure 2). Varroa data collected on 26 October right after Apivar treatment was omitted from the dataset and excluded from statistical analysis.

### 3.3. Viral and Nosema Infections

**Figure 3:**
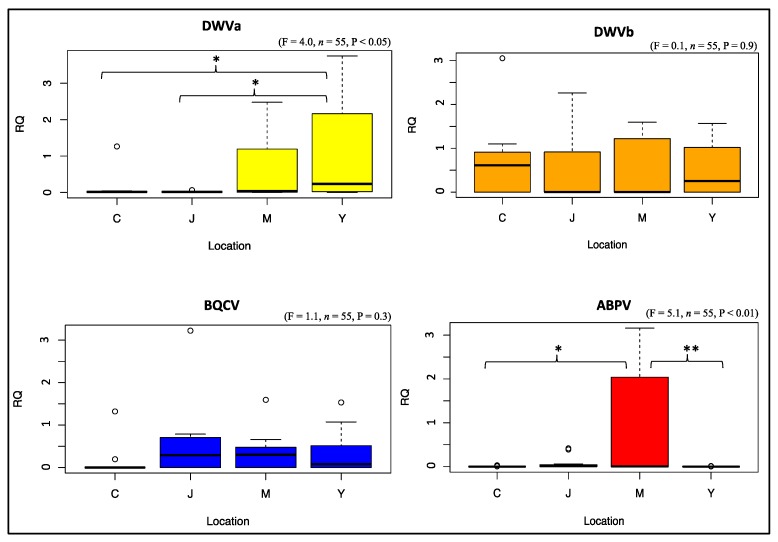
Relative quantification (RQ) of overtime viral infections of the studied colonies exposed by location. Abbreviation codes are (C) Chickasaw, (J) Jackson, (M) Milan, and (Y) Yum-Yum. The Boxplots (aka, Box and whisker Plots) summarize the data distribution based on minimum, first quartile, median, third quartile and maximum values. Viruses are: deformed wing virus type a and b (DWVa&b), black queen cell virus (BQCV) and acute bee paralysis virus (ABPV). ANOVA signification levels are * *p* < 0.05, ** *p* < 0.01.

**Figure 4:**
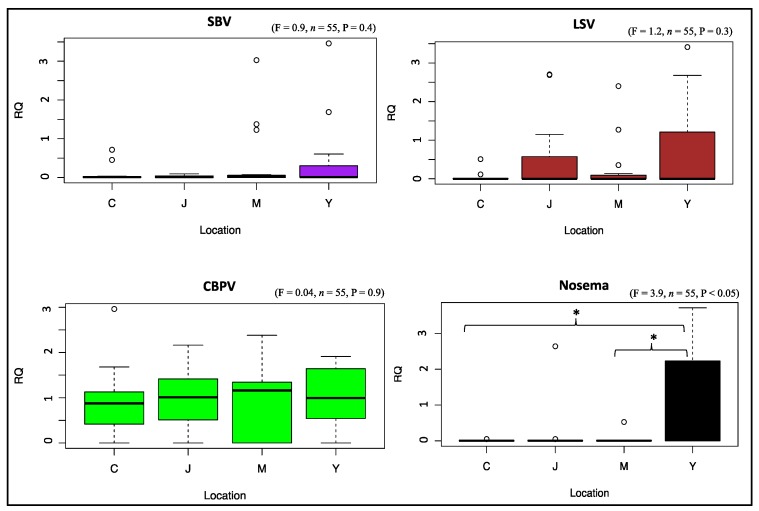
Relative quantification (RQ) of overall viral infections of the studied colonies exposed by location. Abbreviation codes are (C) Chickasaw, (J) Jackson, (M) Milan, and (Y) Yum-Yum. The Boxplots (aka, Box and whisker Plots) summarize the data distribution based on minimum, first quartile, median, third quartile and maximum values. Viruses are: sac brood virus (SBV), Lake Sinai virus (LSV), and chronic bee paralysis virus (CBPV). Levels of nosema are also given for colonies of each location. ANOVA signification levels are * *p* < 0.05.

In total, 96 honey bee samples were analyzed by RT-qPCR for viral and nosema infections. Eighty samples of the 96 were successfully analyzed (RNA extraction and RT-qPCR amplification). Based on the normalization factor values of the four reference genes used in our amplifications, only samples that had a normalization factor >0.5 were selected and statistically treated. This threshold guarantees selection of samples with high accuracy for both RNA quality and amplification. As a consequence, the analytical and statistical results of the honey bee pathogens were based on 55 final samples. Overall, DWVa was significantly higher (*p* < 0.05) in colonies of Milan and Yum-Yum than those of Jackson and Chickasaw (Figure 3). Similarly, infection with ABPV was significantly greater (*p* < 0.01) in Milan’s colonies than colonies of the other three locations (Figure 3). No other significant differences were observed for viral infections between colonies of different locations (Figure 4). However, nosema infections were significantly higher (*p* < 0.05) in Yum-Yum compared with Chickasaw and Milan (Figure 4). Regarding the AG area groups, no significant differences in infection of any of the studied viruses or nosema were recorded (Figures S1 and S2).

### 3.4. Overall Pathogen and Treatment Effects

Results of the GLM analyses suggest that two locations (Jackson, Yum-Yum) had a highly significant effect on varroa load (Model 1: df = 297, *p* < 0.001; Table 2 followed by Milan (*p* = 0.04). The higher impact of the location/treatment on colony weight was recorded in both Yum-Yum (Model 2: Estim. = 5.74 ± 1.2, df = 315, *p* < 0.001) and Milan (Estim. = 4.65 ± 1.2, df = 315, *p* < 0.001), Table 2. This means according to the model estimation that Yum-Yum and Milan provided (5.7 and 4.6) kg increases in colony weight, respectively. Data of the colony weight was used from our previously published study [26].

Jackson was the only location that had a clear effect on varroa load when all over pathogens were accounted (Model 11: Estim. = 7, df = 84, *p* = 0.004; Table 2). Similarly, when varroa load is studied as a function of the weight and location with or without pathogens, two variables have exclusive impact on varroa load; Jackson location, and colony weight (Model 1, 8, 9, 10: *p* < 0.001, Model 11: *p* = 0.004; Table 2). This indicates that regardless the AG area factor; the viral effect is mostly masked when the weight effect is accounted.

### 3.5. Pesticide Residues

#### 3.5.1. Forager Bees

The results of the forager bees of each location showed some residues of different pesticides (Table 3). Concentrations (3 ppb) of imidacloprid (neonicotinoid) were identified in foragers of Apiary 2. Some other low concentrations of herbicide and fungicide were recorded on Apiary 3’s foragers (Table 3).

#### 3.5.2. Honey and Wax

Honey samples did not show pesticide residues except some very low concentrations of fluvalinate (Acaricides) in Apiaries 2 and 3. However, wax samples contained much more diverse residue of chemical substances, mostly acaricides (Table 3). None of those concentrations exceeded the bee oral LD_50_. Besides acaracides, 3.7 ppb of imidacloprid were found in wax of Chickasaw’s colonies (Table 3).

#### 3.5.3. Winter and Dead Bees

One compound was identified in bodies of the winter bees (Dimethylphenyl Formamide DMPF) of all location at various concentrations. At two locations (Jackson and Milan), high concentrations of several pesticides were found in dead honey bees collected from the boards placed in front of hive (Table 3). Chemical analyses of the dead bees revealed concentrations of neonicotinoids (imidacloprid and its metabolite, clothianidin, and thiamethoxam) ranging from (3.3 ppb) to extremely high level (623 ppb) (Table 3). High concentrations of carbaryl (107 ppb) and methamidophos (14 ppb), both insecticides, were also identified in the dead bees collected from the hives’ entrances.

#### 3.5.4. Crop Flowers

Various pesticide residues were found on the crop flowers. Neonicotinoids were recorded on cotton flowers at both the Milan and Yum-Yum locations at 25 ppb (imidacloprid) and 24 ppb (thiamethoxam), respectively (Table 3). Acephate was also detected in Jackson (309 ppb) and Milan (4190 ppb) as well as other insecticides including bifenthrin and methamidophos, a metabolite of acephate (Table 3). Imidacloprid was found at relatively low concentrations (5.3 and 2.4 ppb) on soybean flowers from the Jackson and Yum-Yum, respectively. Sorghum, bifenthrin, spinosad, and several other pesticides were identified at the Jackson and Milan locations. Other detected pesticides residues are shown in Table 3.

## 4. Discussion

Varroa mites, in addition to other stressors, are known to be one of the main causes of colony loss [5,6]. The relatively large number of varroa detections (20 observations) taken on the 16 experimental colonies that were subjected to different landscapes and potential exposure to pesticides provided robust results. Varroa infections exposed by colony and date mostly indicated that hives with bigger populations were likely to have higher varroa loads (H4, H8, and H9) than weaker ones (Figure 1). Colonies located in AG areas, with potentially higher risk of exposure to pesticides, exhibited significantly higher (F = 41.9, *n* = 301, *p* < 0.001) varroa infestation than those of the NAG areas (Figure 2). These data are in agreement with previously published studies testing the effects of exposure to pesticides on varroa loads [31,32]. However, contradictory results were recently obtained while studying the effect of clothianidin-dressed oilseed rape on honey bees, showing no difference in varroa infestation in hives of insecticide treated and untreated fields [45]. Thus, it is not clear whether the higher varroa loads recorded in the AG areas (Jackson, Milan, Yum-Yum; Figure 2) of our study were a consequence of colony size, exposure to pesticides, or both. This inability to uncouple factors is very common in ecological and in situ studies, especially where no organic crop fields are available to be assigned as control treatment. In order to overcome and investigate this further, we performed correlation analyses between the varroa loads and the colony weights (Figure S3A,B). Varroa load positively correlated with the colony weight in almost all colonies except for those of the NAG area, (Figure S3A,B; colony weights used in these correlations were previously published [26]). In other words, it is more likely that higher varroa loads recorded in the AG areas are because of the AG areas’ bigger population sizes comparing with those of the NAG area. Moreover, the results of the GLM models explaining the varroa load as a function of the colony weight and the treatment were in agreement with the previous varroa/weight correlation as well as with the ANOVA results (Model 1, 8; Table 2). The GLM analysis also showed that all AG locations (Jackson, Milan, and Yum-Yum) significantly contributed, to varying degrees, to the varroa load (Model 1; Table 2), but when the weight is added as an explanatory variable to Model 1, only the Jackson location had significantly higher varroa loads, by about 6.61 varroa. The model prioritized the weight effect on both other locations (Milan and Yum-yum) and showed that a 1 kg increase in the colony weight leads to an increase of about 0.48 varroa per colony (Model 8, Estim. = 0.48, *p* < 0.001; Table 2). This indicates that despite the Jackson location having the highest impact on varroa loads (possibly because of high urban activity), weight gain showed strong causal links with varroa load, an association that is still significant even when all other pathogens are accounted for in the other GLM models (Model 8, 9, 10, *p* < 0.001; Table 2). However, that the contribution of pesticide exposure to increased varroa loads by impairing bee immune system [2,29] cannot totally be excluded, as apparently lethal doses of pesticides were identified in dead bees collected from AG areas (Table 3). Despite relatively high concentrations of several insecticides found on the flowers of some crops, low concentrations of only a few pesticides were found in either the hive products (honey and wax) or the adult bees (foragers and winter bees) (Table 3). The pesticide residues found in foraging bees were not based on a single sampling time point but on a pool of robust number of samplings (five times ~1000 foragers/sample) collected from May through October (Table 1). Thus, it seems likely that honey bees were neither routinely exposed to lethal doses of pesticides while foraging in AG or NAG areas (Table 3), nor were agricultural pesticides accumulating within foragers, wintering bees, or honey at levels expected to affect hive health. However, several observations where dead bees were collected in front of hives indicated that foraging bees were occasionally exposed to lethal doses of insecticides in the AG areas, almost certainly from foliar pesticide application that occurred in those locations (Table 3). The high residues of neonicotinoids—in particular imidacloprid (and its metabolite imidacloprid olfen), clothianidin, and thiamethoxam—found in dead bees at Jackson and Milan (Table 3) were almost certainly the cause of death. Carbaryl and 1-naphthol were found in the dead bees but are rarely used in agriculture crops, and these pesticides may have resulted from urban pest management. Interestingly, pesticide residues found in some crop flowers revealed likely sources of pesticide contamination. For instance, acephate, oxamyl, methamidophos, and bifenthrin were detected in the sorghum flowers of Jackson location (Apiary 1; Table 3). Those pesticides are not labelled for sorghum and were not applied to sorghum at this research center, indicating that these detections were the result of sprayer contamination.

The distribution and consistency among locations of the pesticide residues detected in beeswax clearly point to contaminations originating from the wax foundation. This wax was sampled in early period of the season (May) when foliar pesticide applications are not common for the crops grown in this geography and most the contaminants are rarely (if ever) used in crop fields. Furthermore, the pesticide contaminants detected in the beeswax were similarly identified in hives located in the NAG area (Table 3). The numerous pesticides detected in the beeswax (mostly Acaricides) could potentially result in chronic toxicity (Table 3). Indeed, the total number of pesticide products and fungicides with particular modes of action found in beeswax were recently linked to colony mortality [17]. Acaricides are commonly found in beeswax; 18 different pesticide residues were identified in Belgium beeswax including pesticides that are banned in Europe [5,6], which explain the persistency of those chemical substances in the wax. The bees collected at the end of winter in our experiment contained residues of 2,4 DMPF (2,4-dimethylphenyl-*N*′-methylformamidine) at all locations (Table 3). DMPF is a breakdown product of amitraz [46] that clearly resulted from the fall varroa treatment (October, 2015) using Apivar strips on our colonies (Table 1). There is growing evidence indicating a correlation between residual concentrations of acaricides, including coumaphos, and other insecticides within honey bee colonies with drone survival and reproduction as well as queen weight and activity [16,18,47,48].

Honey bee viral diseases have been linked to varroa infestation, as varroa mites are considered to be the main vectors that transmit and propagate viruses within and among colonies [46,49,50,51,52]. Only two viruses (DWVa and ABPV) significantly varied across locations (Figure 3), and none varied significantly with consideration to treatment factor (AG and NAG) (Figures S1 and S2), indicating that colonies in the AG areas did not show substantially higher viral infection that could be linked to exposure to pesticides. GLM analysis, which accounted ecological and random factors, confirmed this finding for both ABPV in Milan and DWVa in Yum-Yum locations only (Model 3, 6; Table 2). We conclude that the increases of varroa population in the AG areas were not associated with substantial higher viral prevalence. This is very consistent with the GLM analysis, in which no links were established between varroa load and viral infection at any time, but rather both treatment and weight factors seem to be the major players vis-à-vis varroa infestation (Model 9, 10, 11; Table 2). Similarly to the viruses, nosema infection was significantly higher in Yum-Yum location *p* < 0.05, Figure 4) but did not differ on average between AG and NAG areas (Figure S2). Considering the pathogen data as a whole, and from a biological point of view, it appears that none of the viruses studied here reached a level sufficient to cause widespread bee mortality, and that varroa mite populations remained below virulent thresholds across our colonies and locations. This conclusion is further supported by the correlation conducted among all studied pathogens (varroa, virus, and nosema), which showed no remarkable correlations (Figure S3C), as well as from the GLM results in which no significant values were obtain for any pathogen when treated as explanatory variables (Model 9, 10, 11; Table 2).

## 5. Conclusions

Our data suggest that honey bee colonies located and foraging in AG areas exhibit higher level of varroa infestation, not necessarily resulting from exposure to pesticides but rather from larger population size. There is no significant evidence that viral and nosema infections varied among colonies in regard of the landscape and potential exposure to pesticides. Honey bee colonies located in AG areas are subjected to higher risk of lethal exposures to pesticide, mostly because of foliar application in the crops of those areas particularly cotton and sorghum during the year of this study. Honey, the principal source of beekeeper income and the main hive product, was generally free of contamination from agricultural pesticides in both AG and NAG areas. Our data show constant significant effects of Jackson location on varroa load, which could be link to the high urban activity in this area.

## Figures and Tables

**Table 1:** Shows the location of each experimental apiary and the number and date of samplings performed in each apiary. Worker bees were sampled for viral and nosema infections while the rest were used for chemical analysis.

	Apiary 1	Apiary 2	Apiary 3	Apiary 4
Location	Jackson	Milan	Yum-Yum	Chickasaw
Hive Equipment	Varroa mite screen board			
Varroa treatment	Once/October-2015/2 strips of Apivar per hive			
N° of Sampling/Chemical analysis				
Forager	5 times (May to September) 2015			
Worker (Viral)	6 times (May to October) 2015			
Winter bees	One time (March 2016)			
Dead bees	29 July and 17 August 2015	26 June and 27 July 2015	None	27 July 2015
Honey	4 times (June to September) 2015			
Wax	1 time (May 2015)			

**Table 2:** Output results of the generalized linear mixed-effects analyses conducted on the dataset. Only significant results were reported in this table. Respond and explanatory variables as well as the fixed effects used in each model are also reported. In all GLMMs, one random effect was considered; agricultural area (AG area). In last three models, only significant fixed effects are provided. For full GLM outputs, refer to the DOI link in the supplementary information.

Model Number	Response/Explanatory Variable	Fixed Effects	Estimate Value	Std. Error	DF	*T*-Value	*p*-Value
(1)	Varroa~treatment	Jackson	7.21	1.6	297	4.38	<0.001
		Milan	3.12	1.5	297	1.9	0.04
		Yum-Yum	5.74	1.5	297	3.65	<0.001
(2)	Weight~treatment	Jackson	0.4	1.2	315	0.3	0.7
		Milan	4.65	1.2	315	3.9	<0.001
		Yum-Yum	5.74	1.2	315	4.81	<0.001
(3)	ABPV~treatment	Jackson	0.2	71.8	92	0.003	0.9
		Milan	173.9	62.2	92	2.79	0.006
		Yum-Yum	0	58.9	92	0	0.9
(4)	BQCV~treatment	Jackson	110.7	57.8	92	1.9	0.057
		Milan	1.7	50.1	92	0.03	0.9
		Yum-Yum	1.8	47.5	92	0.03	0.9
(5)	CBPV~treatment	Jackson	−83.4	45.9	92	−1.8	0.07
		Milan	−70.9	39.7	92	−1.7	0.07
		Yum-Yum	−82.1	37.6	92	−2.1	0.03
(6)	DWVa~treatment	Jackson	−2.7	283	92	−0.01	0.9
		Milan	44.1	246	92	0.1	0.8
		Yum-Yum	492.2	232	92	2.1	0.03
(7)	DWVb~treatment	Jackson	−102.3	54.4	92	−1.8	0.06
		Milan	−113	47.1	92	− 2.4	0.01
		Yum-Yum	−115	44.6	92	− 2.5	0.01
(8)	Varroa~treatment + weight	Jackson	6.61	1.52	296	4.32	<0.001
		Milan	0.7	1.5	296	0.4	0.6
		Yum-Yum	2.9	1.5	296	1.9	0.053
		Weight	0.48	0.06	296	7.03	<0.001
(9)	Varroa~treatment + weight + all viruses	Jackson	6.21	1.77	87	3.49	<0.001
		Weight	0.53	0.06	87	8.18	<0.001
(10)	Varroa~treatment + weight + all viruses + nosema	Jackson	6.37	1.85	83	3.44	<0.001
		Weight	0.53	0.06	83	7.91	<0.001
(11)	Varroa~treatment + all viruses + nosema	Jackson	7.05	1.64	84	2.90	0.004

**Table 3:** Results of the pesticide residue detection performed by liquid chromatography-mass spectrometry (LC-MS) for dead bees, foragers, honey, wax, and crop flowers from each location. LD_50_ is based on the data provided by [44] and the Ecotoxicology databases of the US Environmental Protection Agency. Level of detection (LOD) varied between 1–50 ng/g.

Sample	Pesticide	Apiary 1 (Jackson) PPB	Apiary 2 (Milan) PPB	Apiary 3 (Yum-Yum) PPB	Apiary 4 (Chickasaw) PPB	LD_50_ Oral (ng/bee)
Dead Bees	Imidacloprid	3.3	190	NA		13
	Imida. Olfen		623	NA		28
	Clothianidin	43	70	NA		4
	Thiamethoxam		146	NA		5
	Carbaryl		107			150
	Methamidophos		14.3			200
	1-Naphthol (carbaryl)		230			10,500
Foragers	Imidacloprid		3.1			13
	Azoxystrobin ^1^			7		25,000
	Pendimethalin ^2^			111		665,000
Winter Bees	DMPF (amitraz)	63.2	64.8	150	115	750,000
Honey	Fluvalinate ^3^		5.4	5		45,000
Wax	Imidacloprid				3.7	13
	Fluvalinate ^3^	103	122	146	205	45,000
	Dicofol ^3^	4.3	8	11	8	10,000
	Carbendazim ^1^	5	5	5	5	50,000
	Coumaphos	6	5	5	6	4600
	Fenpyroximate ^3^	5	5	5	8	1100
	Metalaxyl ^1^				7	269,000
	Atrazine ^2^		8	10		1000
**Crop flowers**						
Cotton	Imidacloprid		25		NA	13
	Thiamethoxam			24	NA	5
	Acephate	309	57	4190	NA	230
	Bifenthrin	91			NA	200
	Cyhalothrin	4			NA	22
	Methamidophos	30.2	6504	1300	NA	200
	Oxamyl ^4^		851	271	NA	380
Soybean	Imidacloprid	5.3		2.4	NA	13
	Azoxystrobin ^1^	44			NA	25,000
	Fenpyroximate ^3^			5	NA	11,000
	Metolachlor ^2^			191	NA	1,260,000
	Pyridaben			10	NA	550
Sorghum	Acephate	124			NA	230
	Cyhalothrin	187	35		NA	22
	Methamidophos	23			NA	200
	Azoxystrobin ^1^	15	17		NA	25,000
	Atrazine ^2^			8	NA	1000
	Chlorpyrifos	3.2			NA	130
	Bifenthrin	186			NA	240
	Oxamyl ^4^	5			NA	380
	Spinosad		108		NA	57
	Pyraclostrobin ^4^		286		NA	73,000
Corn	Metribuzin ^2^	9			NA	567,000

^1^ Fungicide, ^2^ Herbicide, ^3^ Acaricide, ^4^ Insecticide and nematicide, the rest are insecticides. (NA) means no sample analyzed.

## Supplementary Materials

The following are available online at http://www.mdpi.com/2075-4450/9/2/65/s1. Figure S1: Relative quantification (RQ) of overall viral infections (DWV, BQCV, ABPV) exposed by AG areas; Figure S2: Relative quantification (RQ) of overall viral infections (SBV, LSV, CBPV) exposed by AG areas; Figure S3: (A) Correlation matrixes between varroa infestation and colony weight exposed per colony and (B) AG areas. (wh1) to (wh16) are the colony weights and (vh1) to (vh16) are the colony varroa loads. (C) Correlation matrix of overall pathogen infection (varroa mite, viruses and nosema) of the 16 studied colonies; Figure S4: Shapiro-Wilk tests and variable distribution and normalization for both varroa and weight variables. Relevant data and supporting materials for this study (e.g., full list of analytical reports, RT-qPCR primers, and photos) are made available on the LabArchives’ website under the DOI: http://dx.doi.org/10.6070/H44T6GZK.
