# Supplementary material for: Honey Bee Survival and Pathogen Prevalence: From the Perspective of Landscape and Exposure to Pesticides
Source: Insects. 2018 Jun 13;9(2):65. doi: 10.3390/insects9020065 (PMC6023357; doi:10.3390/insects9020065)
Supplement: Supplementary file 1 [file insects-09-00065-s001.pdf]

Fig. S1

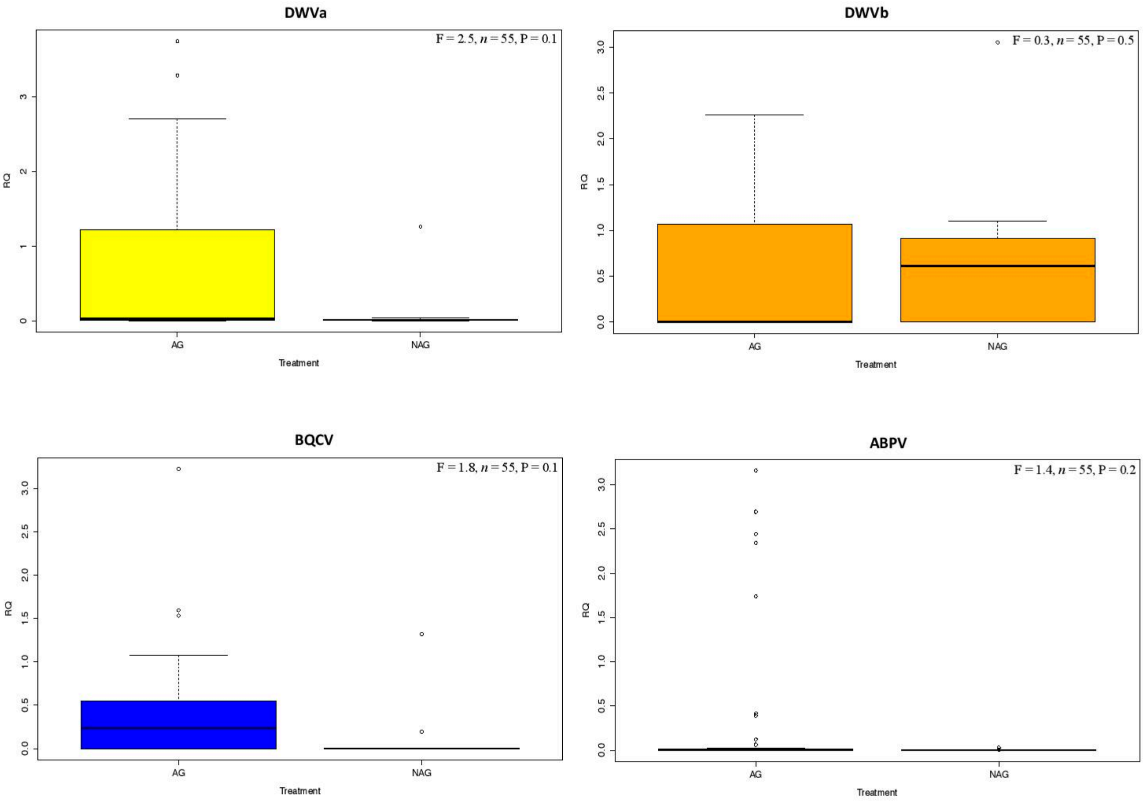

Figure S1: Relative quantification (RQ) of overall viral infections of the studied colonies exposed by AG areas. The Boxplots (aka, Box and whisker Plots) summarize the data distribution based on minimum, first quartile, median, third quartile and maximum values. Viruses are: deformed wing virus type a and b (DWVa,b), black queen cell virus (BQCV) and acute bee paralysis virus (ABPV). ANOVA showed no significant differences between groups.

Fig. S2

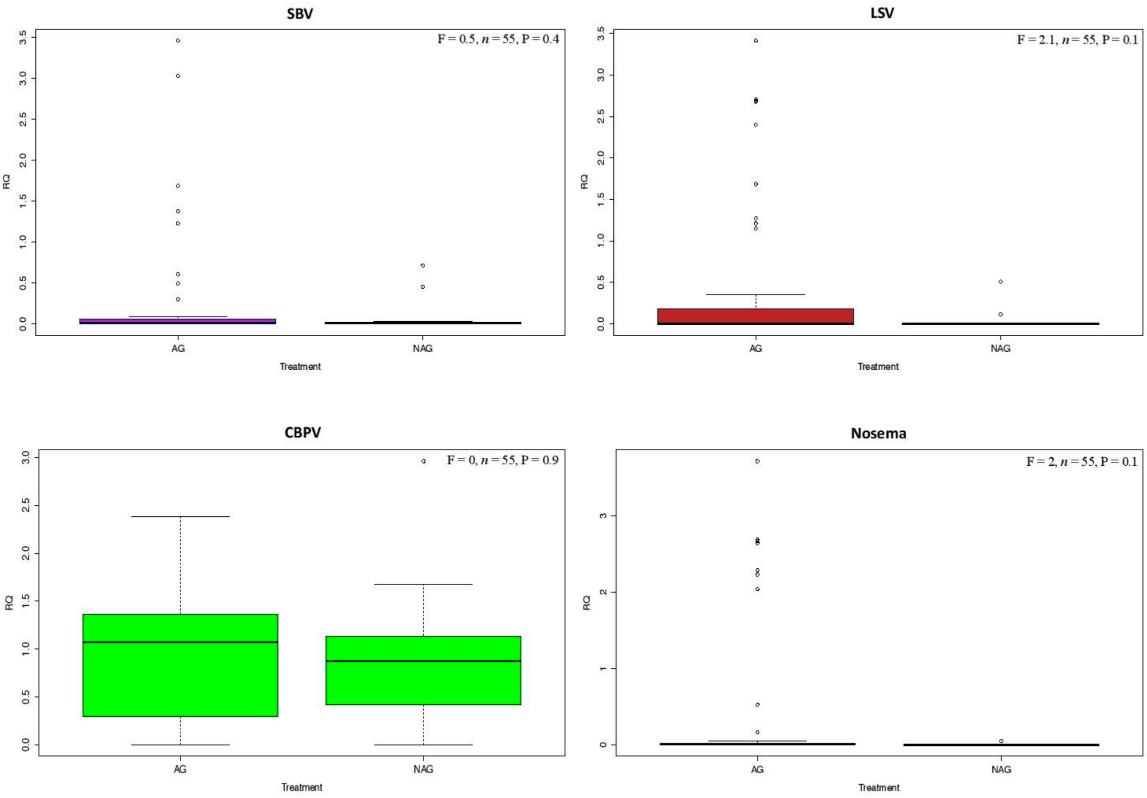

Figure S2: Relative quantification (RQ) of overall viral infections of the studied colonies exposed by AG areas. The Boxplots (aka, Box and whisker Plots) summarize the data distribution based on minimum, first quartile, median, third quartile and maximum values. Viruses are: sac brood virus (SBV), Lake Sinai virus (LSV) and chronic bee paralysis virus (CBPV). Level of nosema infections are exposed by AG area. ANOVA showed no significant differences between groups.

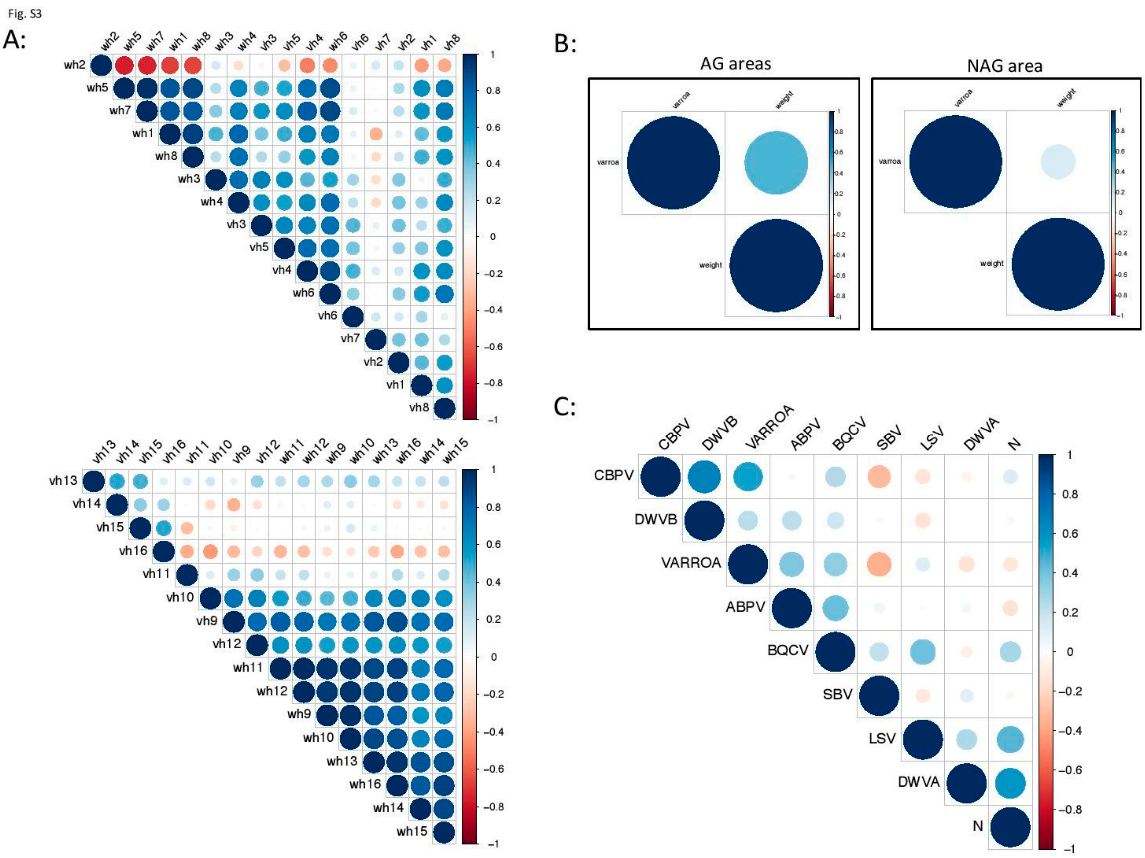

Figure S3: (A) Correlation matrixes between varroa infestation and colony weight exposed per colony and (B) AG areas. (wh1) to (wh16) are the colony weights and (vh1) to (vh16) are the colony varroa loads. (C) Correlation matrix of overall pathogen infection (varroa mite, viruses and nosema) of the 16 studied colonies.

Fig. S4

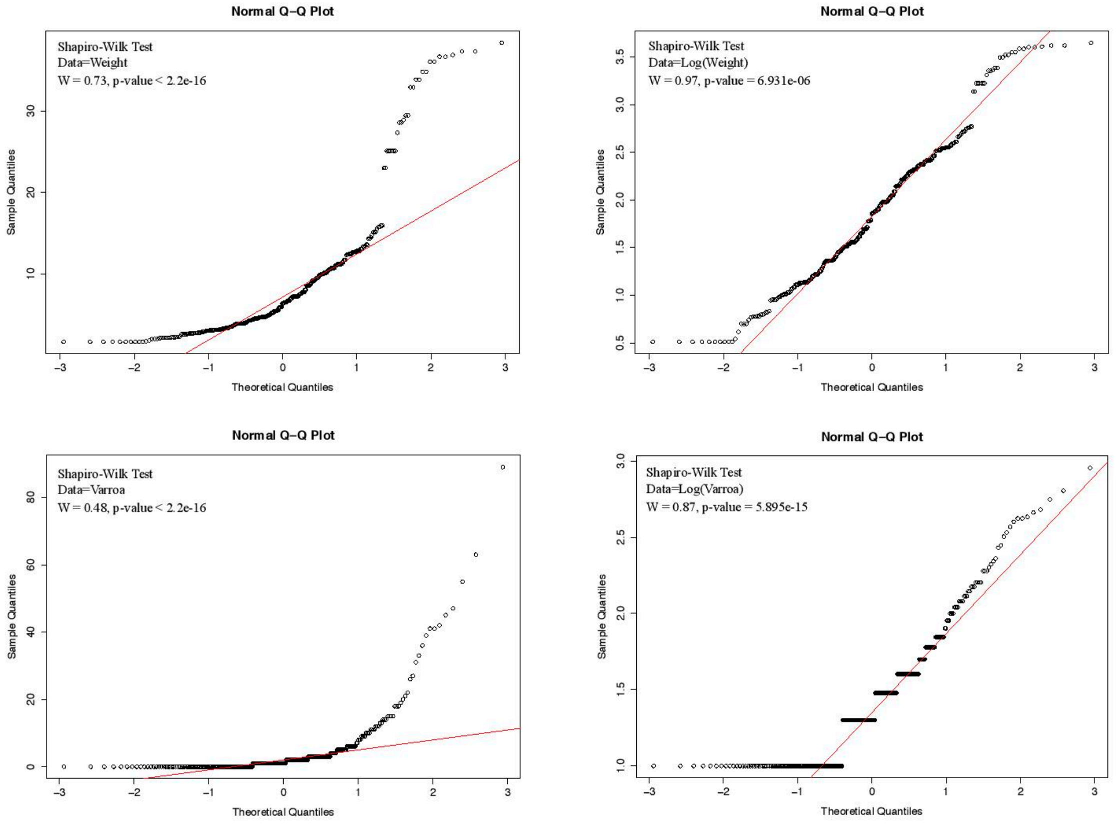

Figure S4: Shapiro-Wilk tests and variable distribution and normalization for both varroa and weight variables.
